# TiO_2_/S-Doped Carbons Hybrids: Analysis of Their Interfacial and Surface Features

**DOI:** 10.3390/molecules24193585

**Published:** 2019-10-05

**Authors:** Teresa J. Bandosz, Alfonso Policicchio, Marc Florent, Po S. Poon, Juan Matos

**Affiliations:** 1 Department of Chemistry and Biochemistry, The City College of New York, New York, NY 10031, USA; alfonso.policicchio@gmail.com (A.P.); mflorent@ccny.cuny.edu (M.F.); 2 Unidad de Desarrollo Tecnológico (UDT), Universidad de Concepción, 403000 Concepción, Chile; p.poon@udt.cl; 3 Millennium Nuclei on Catalytic Processes towards Sustainable Chemistry (CSC), 7810000 Santiago, Chile

**Keywords:** S-doped carbon, TiO_2_, hybrids, surface chemistry, porosity, interface

## Abstract

Hybrids containing approximately equal amounts of P25 TiO_2_ and S-doped porous carbons were prepared using a water-based slurry mixing method. The materials were extensively characterized by adsorption of nitrogen, potentiometric titration, thermal analysis in air and in helium, XRD, XPS and SEM. The collected results showed the significant blockage of carbon micropores by TiO_2_ particles deposited on their outer surface. The formation of a new interface, especially for the S-rich samples, might also contribute to the porosity alteration. Analysis of surface chemistry suggested the presence of Ti-S bonds with an involvement of sulfur from thiophenic species in the carbon phase. The latter, especially when polymer-derived, was mainly deposited on the TiO_2_ nanoparticles. Formation of Ti-S stabilized sulfur and increased the ignition temperature of the hybrids, especially those with a high content of sulfur, in comparison with the ignition temperature of carbons. The surfaces of hybrid with S-containing carbons was also thermally very stable and of basic chemical nature. The formation of interfacial structures Ti-C was detected by XPS analysis suggesting a partial reduction of the Ti.

## 1. Introduction

One of the major targets of the XXIst century is to find efficient methodologies for environmental remediation and this includes the treatment of polluted water. Water pollution is not only linked to industrial activities, but it is also the result of the exponential increase of the world population, expected to reach more than 9.5 billion by 2045. The appearance of new micropollutants, also called emerging pollutants [1,2], is a major problem [3] that is related to the complexity of the chemical matrix associated with residual domestic waters. Among several advanced oxidation processes, heterogeneous photocatalysis based on photoactive semiconductors such as TiO_2_ is a common approach [4,5,6,7]. An extensive description of the surface chemistry parameters affecting the photocatalytic activity of TiO_2_, including the doping effects and the formation of interfacial junctions, among others, has been reported by Puga [8]. This technology is highly efficient, sustainable and low-cost. However, besides the classical limitations of TiO_2_ related to its photoactive performance such as the low activity under visible light, the high recombination rate of photogenerated electron–hole pairs, and the low stability in terms of their recovery and reutilization [9], this material has been recently listed as a suspected carcinogen to humans [10]. Thus, alternative photocatalysts are required for the treatment of polluted water.

To improve the performance of TiO_2_ and to partially overcome its limitations, heterostructures of this semiconductor with nanoporous carbons have been investigated [11,12,13,14,15,16,17,18,19,20,21,22]. They have the potential to provide efficiency, biocompatibility and low cost, which are important features for the solar-driven treatment of polluted water. The remarkable photoactivity of nanoporous carbons have been attributed both to a tailored pore size distribution and the relative ease of chemical functionalization of their surface [23]. It has been found that heteroatom-containing groups on the surface of nanoporous carbons, mainly O-containing groups, are responsible for the formation of reactive oxygen species (ROS) such as hydroxyl radical (^•^OH) and superoxo anion radical (O_2_^•^¯) [14,16,17]. By contrast, only limited works have reported the role of reactive sulfur species (RSS) in the photocatalytic activity of nanoporous carbons [15,24,25,26,27]. Several works have reported interesting features concerning to the interfacial chemistry and porosity characteristics in carbon/TiO_2_ composites [20,21,22,26,27,28,29,30,31,32,33,34]. These include O-, N-, P- and S-containing nanoporous carbons-TiO_2_ composites which have exhibited an enhancement in the photocatalytic degradation under solar irradiation of different types of organic molecules such as methylene blue [12,29,32], imidacloprid [13], 2,4-dichloroacetic acid [13], 4-chlorophenol [28], bisphenol [33], and phenol [13,26,27,30]. It has been found that besides micropores, a contribution of ca. 10–20% mesopores to the total pore volume is required for the efficient mass diffusion of pollutant molecules from the bulk solution [12,13,31,33]. Moreover, the formation of interfacial Ti-heteroatoms structures can modulate the photoactivity of TiO_2_ [20,21,22,26,27,28,34]. Gomis-Berenguer and co-workers [26] have shown that the photochemical conversion of aromatic molecules such as phenol inside the pore framework of nanoporous carbons is very sensitive to the nature of the S-containing groups. These authors showed that the photochemical reactivity can be enhanced by tailoring the pore size distribution and the types of O- and S-containing surface groups. In a very recent work [27], our group has also reported that specific S-C groups on porous carbon surfaces enhanced the photoactivity and governed the selectivity of products.

Since the previous state-of-the -art suggests that both the interfacial chemistry and the porosity of the heteroatoms-containing carbon-based/TiO_2_ composites play an important role in the photoefficiency of the semiconductor, and also that the changes in the intermediates formed during phenol degradation, mainly in the type of dihydroxybenzenes, are commonly associated with changes in the interfacial structures of TiO_2_-based photocatalysts [35], the objective of this work is the evaluation of the surface features of binary S-doped nanoporous carbons/TiO_2_ heterostructures, with an ultimate goal to use them for a solar light-driven photocatalytic degradation of aromatic pollutant molecules such as phenol. Therefore, this paper focuses on the influence of porosity and chemistry of these hybrids. The emphases are on the newly formed interface and on the possible effect of these new surface features on the target application as photocatalysts. Even though it has been reported previously that the interfacial formation of polythiophene-TiO_2_ structures is able to modulate the optical properties of TiO_2_ [36], no detailed studies of S-doped nanoporous carbon-TiO_2_ composites materials have been conducted. Based on recently addressed visible light photoactivity of S-doped carbons [26,27], we have assumed that building TiO_2_/S-doped hybrids be beneficial for solar light driven applications, such as advanced oxidation process or solar light harvesting in general.

## 2. Results and Discussion

The carbons used to build the hybrids have been described in detail elsewhere [27]. For the sake of discussion, it is important to mention that the treatment with H_2_S increased the amount of sulfur on the surface and its content in BAX, BAX-S, C1, C1-S, C2, C2-S was 0, 0.3, 0.5, 1.3, 1.6 and 1.8 at %, respectively, with over 80% of sulfur in bisulfide/thiophenes in C1-S and C2-S.

**Figure 1:**
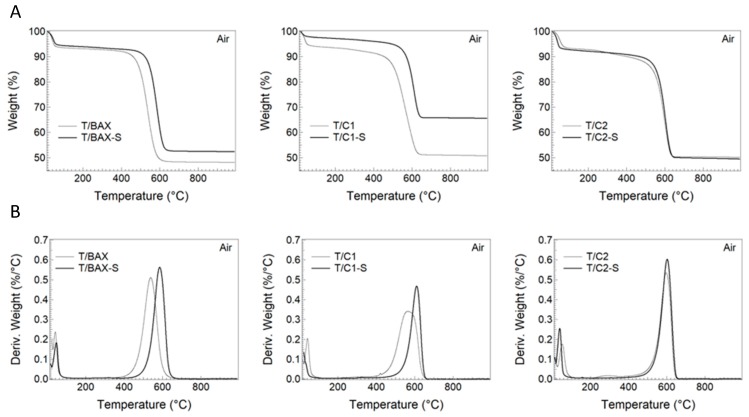
(**A**) TG and (**B**) DTG curves in air for the hybrids studied.

To evaluate the bulk composition of the hybrids, TA experiments were run in air and the residue left after burning is considered as representing TiO_2_. The TG curves are collected in Figure 1A. They show that the content of TiO_2_ in all samples but in T/C1-S is about 50 ± 2%. In T/C1-S there is 65 ± 1% of TiO_2_. The smaller amount of carbon in T/C1-S is a consequence of C1-S carbon TiO_2_ interactions. Apparently, this carbon phase did not “blend/mix” homogenously with TiO_2_. The experiment showed that it had a high affinity to interact with the beaker and filter materials as a separate entity. This might be a consequence of this carbon specific surface chemistry. As discussed in [27], it has the very small (5.5 at % and the smallest among all carbons used to build the hybrids) content of oxygen, which likely limits the interactions of this phase with titania. Interestingly, building the hybrids with S doped carbons increased the ignition temperature of about 50 °C for T/BAX-S and T/C1-S compared to the non S-containing samples (585 °C for T/BAX-S vs. 538 °C T/BAX and 610 °C for T/C1-S vs. 564 °C for T/C1; Figure 1B). On the other hand, no effect was found for T/C2-S (602 °C for T/C2-S vs. 597 °C for T/C2). This shift in the ignition temperature might be related to the effect of sulfur, since its amount visibly increased for C1-S and BAX-S. For C2-S only a slight increase in the initially high content of sulfur in C2 was found.

Another plausible explanation might be the formation of new interface chemistry between TiO_2_ and the C2 carbon series, which had the highest sulfur content. This increase in the ignition temperature is an interesting observation, since sulfur has not been known as increasing the thermal stability of carbons when doped into their matrices. For BAX, BAX-S, C1, C1-S, C2 and C2-S the measured ignition temperatures were 537, 588, 575, 609, 601 and 602, respectively. This suggests that the ignition temperatures of the hybrid are governed by the ignition temperatures of carbons.

**Figure 2:**
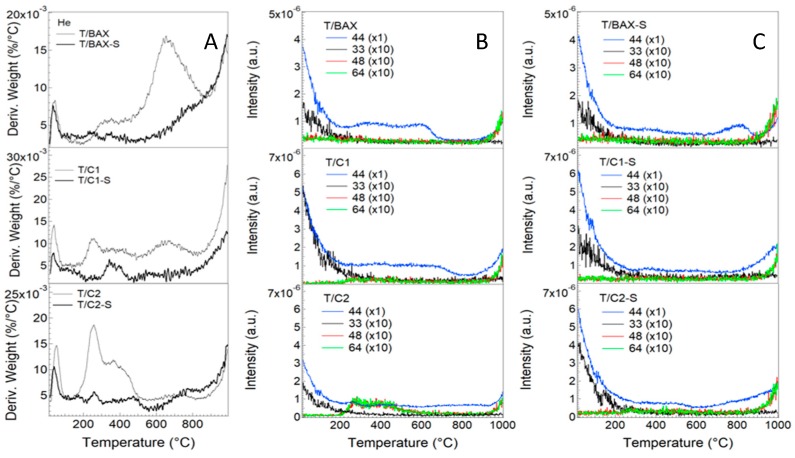
(**A**) DTG curves in helium; (**B**) *m*/*z* thermal profiles for the initial samples; (**C**) *m*/*z* thermal profiles for the H_2_S-treated samples.

The DTG curves in helium and arbitrary chosen *m*/*z* thermal profiles for 44 (CO_2_), 33 (HS^−^), 48 (SO) and 64 (SO_2_) are collected in Figure 2. Generally, the samples were thermally very stable and T/BAX, T/BAX-S, T/C1, T/C1-S, T/C2, T/C2-S lost only 8.2, 5.2, 8.9, 4.0, 7.6 and 5.1%, respectively, upon heating to 1000 °C. TiO_2_ in this temperature range lost 3% of its weight. Remarkably, all hybrids formed with H_2_S-modified carbons showed higher thermal stabilities than those formed with the initial carbons. Since the weight loss was really small, *m*/*z* thermal profiles do not reveal too much information. Decomposition of oxygen containing groups is demonstrated by the release of CO_2_, especially from non-H_2_S-treated samples and some sulfur is released from T/C2 between 200–600 °C, likely from sulfoxides and sulfones [37]. Sulfur in other samples, if present, is very stable and do not decompose even at high temperature and only small amount of CO_2_ is detected in the off-gases.

**Figure 3:**
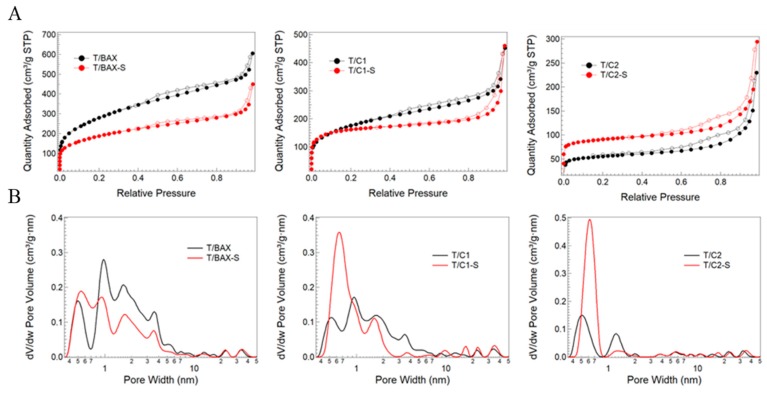
(**A**) Nitrogen adsorption isotherms; (**B**) Pore size distributions.

The addition of carbon to TiO_2_ is expected to provide an additional surface area/pore volume from the carbon phase, which might benefit an adsorption process during the photocatalytic activity. Textural parameters were calculated from the measured nitrogen adsorption isotherms (Figure 3A). From them also pore size distributions, PSDs (Figure 3B) were derived using NLDFT [38]. Interestingly, the effect of the TiO_2_ addition on the development of mesopores (TiO_2_ is mesoporous, with a surface area of 45 m^2^/g, and with a negligible volume of micropores, and the volume of mesopores 0.151 cm^3^/g [13]) is very strongly seen for the hybrids with C2 and C2-S. The marked increase in the amount of N_2_ adsorbed at p/p_o_ about 0.9 and distinguished hysteresis loops visible on their isotherms indicate a significant contribution of mesopores in their structure. The volume of mesopores for T/BAX, T/BAX-S, T/C1, T/C1-S, T/C2 and T/C2-S are 0.62, 0.47, 0.49, 0.48, 0.39, and 0.33 cm^3^/g, respectively, and the corresponding degrees of mesoporosity (V_mes_/V_t_) are 0.69, 0.70, 0.73, 0.69, 0.82 and 0.73, respectively. 

The T/C1 and T/C1-S differ in the shape of isotherms owing to more TiO_2_ in the latter sample, where titania predominates the adsorption behavior.

**Figure 4:**
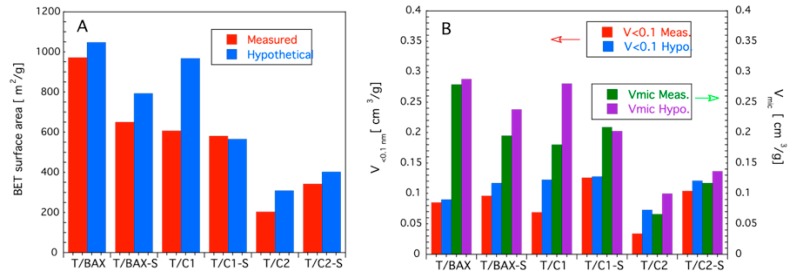
Comparison between the measured and hypothetical values: (**A**) surface area; (**B**) pore volumes of the hybrids.

The comparison of the measured surface areas and volumes of pores smaller than 1 nm and micropores to the hypothetical values calculated assuming the physical mixtures of carbons and TiO_2_ (based on their content and on the parameters of the pore structure of each phase) is presented in Figure 4. As reported elsewhere [13,28], the measured porosity of the heterostructures usually decreases when compared to the hypothetical values calculated assuming a physical mixture. For example, the surface area of a nanoporous carbon-TiO_2_ composite was ca. 123 m^2^ g^−1^ that was ca. 9% lower than the hypothetical value (135 m^2^ g^−1^) considering a weight relation 10:1 for TiO_2_:C [13]. This trend has been also found for more than twenty different TiO_2_-C composites [28]. The latter work suggested that interfacial structures formed between the O-containing groups on the surface of nanoporous carbons and TiO_2_, and found using XPS, XANES, and NEXAFS analyses, might be responsible for this decrease in the surface area and also for other changes in the structural and optical properties of TiO_2_. Moreover, it was also shown that the type of interfacial structures formed was highly dependent on the surface pH of the nanoporous carbons, and therefore, dependent on the type of heteroatom-containing groups in the nanoporous carbons [28].

The decrease in the surface area is also the general effect found for some of our hybrids and it suggests some interfacial interactions or blocking the carbon pores by small TiO_2_ particles. While the porosities of the hybrids with sulfur-free BAX and C1-S resemble the physical mixtures, a biggest decrease in the porosity was found for the C2 series. The high surface area of the T/C2-S compared to that of the T/C2 is a consequence of the marked increase in the area of C2-S compared to that of C2, which was linked to activation during the H_2_S treatment, as discussed in details in Ref. [27].

Although no direct trend for all materials could be established, the hybrids with C1 and C2 carbons which were not exposed to H_2_S treatment and thus have a small sulfur content (from the precursor polymer) and which were most hydrophilic [27], depart most in their porosity from the hypothetical mixtures. Thus S_BET_, V_<0.1 nm_ or V_mic_ for T/C2 are smaller 52%, 115%, and 51%, respectively, from those for hypothetical mixtures. For T/C1, these values are 59%, 78% and 56% smaller, respectively, than the hypothetical ones. For T/BAX the deviations are less than 8%. This suggests that microstructure/chemistry of the polymer derived carbons affects its interactions with TiO_2,_ and the formed interface might result in blocking the carbon phase porosity. The differences in the porosity, likely related to the effects of sulfur, are seen in pore size distributions, PSDs, presented in Figure 3B. All heterostructures without sulfur are more microporous than those containing S.

**Figure 5:**
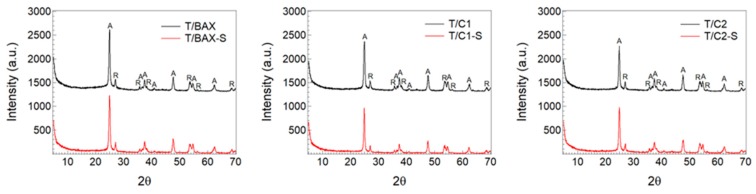
XRD diffraction patterns for the T/C hybrids studied.

The XRD patterns are presented in Figure 5. The integration of all peaks area in the diffraction range 5–75° (based on the diffraction peaks at 2θ = 25.2, 37.8, 48.1, 55.2, 62.7, and 70.2, indexed to the (101), (004), (200), (211), (118), and (220) crystal planes of anatase TiO_2_ (PDF card 84–1286), and to the diffraction peaks at 2θ = 27.5, 36.0, 41.5, 54.0, 57.5, and 68.9, indexed to the (110), (101), (111), (211), and (301) crystal planes of rutile TiO_2_ (PDF card 88–1175, JCPDS)) led to the anatase percentage of contribution [13,39] based on specific peaks (Figure 5) of 77%, 76%, 75%, 75%, 75% and 73% for T/BAX, T/BAX-S, T/C1, T/C1-S, T/C2, T/C2-S, respectively. These values confirmed that anatase/rutile ratio remains constant for all samples but for those with the C-2 series of carbon. A slight decrease in the contribution of anatase, especially for the C2 series after sulfidation was found. Since this carbon has the highest sulfur content and most sulfur in the reduced form, the results suggest that C-S groups interact with the anatase phase and oxygen vacancy in its crystalline structure might promote this [13]. Similar trend of interaction was found between the carbon basic groups and anatase [9,13,28].

**Figure 6:**
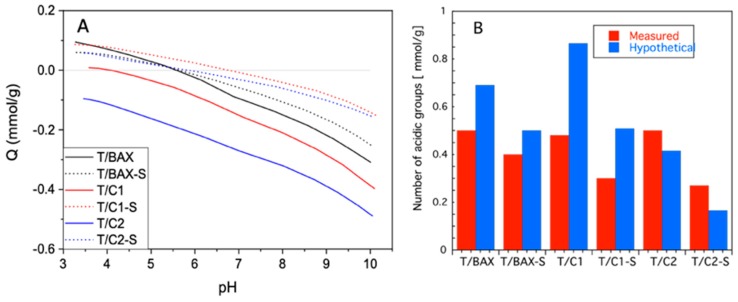
(**A**) Proton binding curves; (**B**) Comparison of measured and hypothetical numbers of surface groups.

Potentiometric titration data is collected in Figure 6. The average surface pH values are similar than those of corresponding carbons [27] and they are 5.97, 5.50, 4.42, 6.86, 3.72 and 5.02 for T/BAX, T/BAX-S, T/C1, T/C1-S, T/C2, T_/_C2-S, respectively. For titania 0.13 mmol g^−1^ of acidic groups were detected in our experimental window. As in the case of the porosity, the measure and hypothetical number of acidic groups for physical mixture was compared. For all hybrid samples the number of detected acidic groups does not follow the composition of the hybrids, assuming the components’ physical mixtures with no interface interactions involved. Generally, for the T/BAX and T/C1 series less groups are detected, and the biggest discrepancy is found for T/C1. The hybrids with S-doped carbons are less acidic (have less groups on the surface) than those with the initial carbons, which follows the changes in the chemical nature of carbons themselves after sulfidation [27].

This process resulted in predominantly basic and hydrophobic surfaces, especially for the C2-S carbon, but when the T/C2 series are considered, the number of groups detected is greater than that hypothetical mixtures. While a decrease can be explained by “screening” of some carbon groups by TiO_2_ owing to the hydrophilic character of the BAX and C1 series [27] an increase in the case of the T/C2 series can be only linked to formation of new acidic group on the interfaces. These results are generally consistent with the trend in porosity analysis.

**Figure 7:**
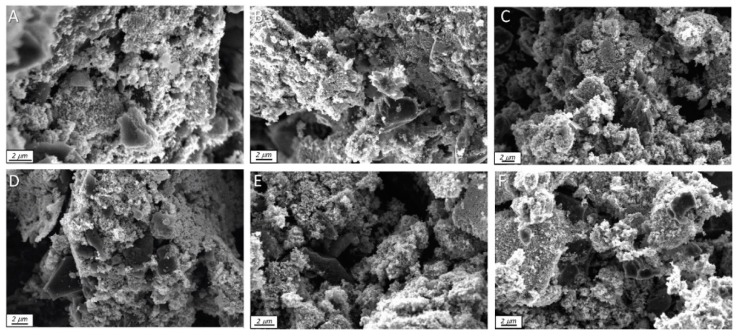
SEM images. (**A**) T/BAX; (**B**) T/C1; (**C**) T/C2; (**D**) T/BAX-S; (**E**) T/C1-S; (**F**) T/C2-S.

To better understand the interface the SEM analysis was carried out. The collected representative images are included in Figure 7. As a general trend, a high degree of dispersion of TiO_2_ nanoparticles on the surface of carbon was observed. No important aggregation of nanoparticles was observed. This result agrees with previous work [40] which reported that nanoporous carbons with hydrophobic surfaces promotes a high dispersion of TiO_2_ nanoparticles through the opposite surface electric charges.

**Figure 8:**
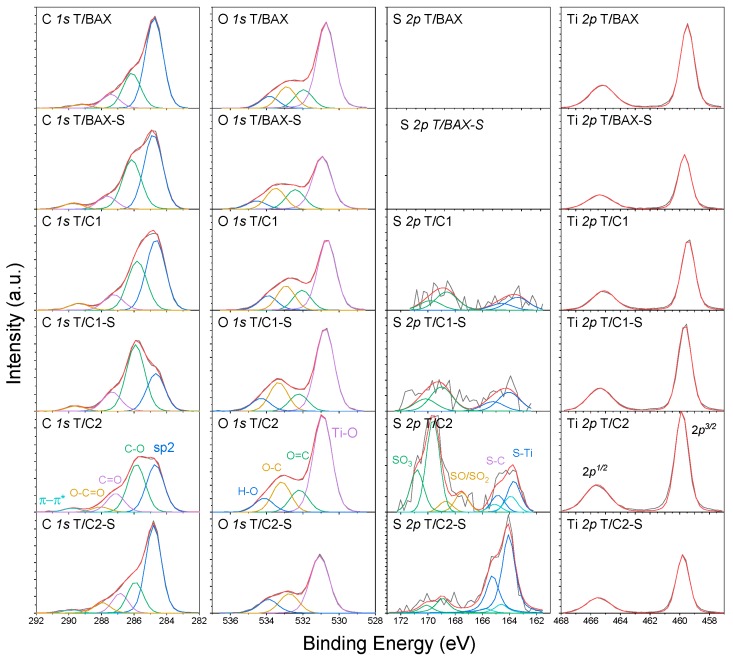
Deconvolution of C 1s, O 1s, S 2p and Ti 2p^3/2^ core energy level spectra. In the case of S 2p, the 2p3^/2^ and 2p^1/2^ contributions (differ by 1.19 eV) are marked with the corresponding colors.

To further investigate the possibility of TiO_2_-C interactions at the interface, XPS analysis was carried out and the results are shown in Figure 8 and collected in Table 1. The smaller amounts of titanium atoms than those of carbon atoms detected on the surface support the hypothesis that carbon is the outside layer. Nevertheless, there is still about 10–20% of all surface oxygen in TiO_2_ on the surface. A high content of oxygen is obviously related to the presence of titania. A rough link of the amount of oxygen to that titania oxygen and subtraction of that amount from the total amount of oxygen atoms leaves the remaining content of oxygen similar to that on the parent carbons [27] for T/BAX-S, T/C1 and T/C2-S. For T/C1-S the carbon phase oxygen content seems much higher than that on the surface of the original carbons, which might be associated with its high content of titania and the different distribution of oxygen. For T/C2-S, interestingly, the content of oxygen in the carbon phase decreased. One must be aware that these calculations are very approximate since some titanium on the surface, although certainly not in majority, could be also in a mixed/less than Ti^4+^ oxidation state as reported elsewhere [28].

The changes in the oxygen content can be also linked to changes in sulfur speciation upon the formation of the hybrids. The content of sulfur in the carbon phase itself was 0.0 0.3, 0.5, 1.3, 1.6 and 1.8 at % for BAX, BAX-S, C1, C1-S, C2, C2-S, respectively [27]. While for the C1 hybrid series the sulfur content decreased about 50%, for the hybrids containing the C2 series that decrease is less pronounced (30% for T/C2 and 11% for T/C2-S). The deconvolutions of S 2p core energy level spectra suggest that in T/C1, T/C1-S and T/C2 there is from 0.15–0.22 at % S (in the absolute amount) in Ti-S bonds. On the surface of the highest sulfur content hybrid, T/C2-S, there is 1.2 at % of sulfur (in absolute amount) in Ti-S bonds. Considering the order magnitude higher content of titania on the surface than that on sulfur that fraction of Ti^+4^ involved in bonds with reduced sulfur species, might remain undetectable.

Interestingly, the highest contributions of Ti-S bonds are found for the samples with carbons having the highest contribution of thiophenic/reduced sulfur (89% in C1-S and 82.2% in C2-S [27]), which suggests that the reduced sulfur has the highest affinity to bind with titanium which might be associated with some level of sulfur oxidation. Indeed, the results show that the contribution of oxidized sulfur in these samples is much higher than on the surfaces of the initial carbons (10.2% of sulfur in sulfones/sulfonic acid in C1-S and 10% in C-2S [27]). Even though such bond formation reaction might not be expected at a room temperature, one must take into consideration that in this particular scenario two photoactive phases are involved. Thus, the photoactivity might promote the detected changes in the chemical environment.

The core energy level spectra of Ti 2p^3/2^ show that Ti^+4^ in oxide is a predominant species. Even though the deconvolution of S 2p suggests the existence of Ti-S bonds, these entities cannot be confirmed by Ti 2p^3/2^ spectra owing to their small absolute amounts, which likely makes them undetectable.

**Figure 9:**
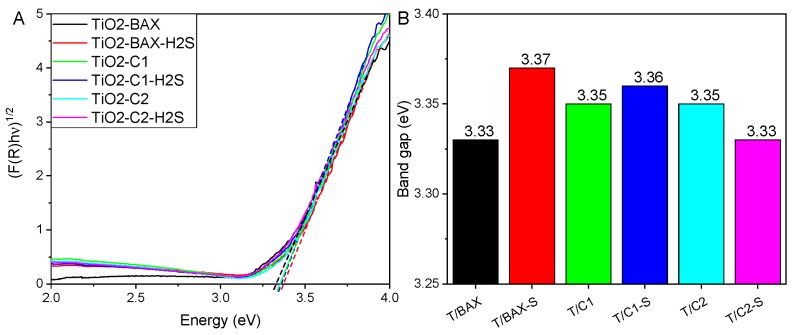
(**A**) Tauc plots for indirect allowed transitions. (**B**) Indirect optical band gaps of the different materials.

To evaluate the effect of hybrid formation on the photoactive properties the absorbance spectra were measured and from the Tauc plots (Figure 9A) the band gaps were estimated (Figure 9B). The addition of carbon slightly increased the band gap, compared to that of TiO_2_ (3.2 eV) supporting the chemical interactions.

No clear trend was found upon the addition of S-doped carbons and only for the C2 series the hybrid with S-doped carbon (C2-S) showed a gap decrease compared to that with C-2. This effect follows the trend in the alteration of anatase content and supports the strong interactions of TiO_2_ surface with carbons having reduced sulfur in C-S bonds incorporated to its matrix. This result agrees with those reported by Puga [8] whom described that in spite of the fact that doping TiO_2_ usually results in a red-shifted absorption edge, this not always guarantees the effective and homogeneous electronic modification of the material, since in some cases absorption can be exclusively caused by isolated sites, not contributing to the bulk electronic bands, as is the case for certain d–d transitions [8].

## 3. Experimental Section

### 3.1. Materials

Commercial wood-based carbon, BAX 1500 (Mead Westvaco, Richmond, VA, USA) and two synthetic carbons, C1 and C2, were used in this study to build the hybrids with commercial TiO_2_ (P25, Evonik, Birmingham, AL, USA). The details in their preparation are included in [27]. Briefly, C1 and C2 were prepared by a direct carbonization of polystyrene sulfonic acid co-maleic acid sodium salt (Mw~20,000, Aldrich, Aldrich, St. Louis, MO, USA) and polystyrene sulfonic acid sodium salt (Mw~70,000, Aldrich) at 800 °C, respectively. Sulfur was introduced to the surface by heating in H_2_S atmosphere (1000 ppm in N_2_) at 800 °C for 3 h. The modified carbons were extensively washed to remove sodium salts and they are referred to as BAX-S, C1-S and C2-S.

TiO_2_:C binary hybrids in 1:1 weight ratio were prepared in situ by the slurry method [13]. TiO_2_ P25 (10 mg) was mixed with of carbon (10 mg) in distilled water (20 mL) at ambient temperature under vigorous stirring for 1 h. Then, the hybrids were filtered and dry at 100 °C for 2h. Apparent compositions of the materials obtained are the effect of both phases’ interactions/hybrid homogeneity levels. The binary photocatalysts are referred to as T/C, were C can be either C1, C2, BAX or S-doped counterparts of these carbons. For the sake of comparison, the relevant surface analyses were also carried out on the P25 TiO_2_. Regarding carbons, for the clarity of presentation, some results included in [27] are reintroduced in the discussion.

### 3.2. Surface Characterization

#### 3.2.1. Porosity and Texture

Nitrogen adsorption isotherms were measured on ASAP 2020 (Micromeritics, Norcross, GA, USA) at −196 °C. The samples were outgassed at 120 °C until a constant vacuum of 10^−8^ Pa was reached. Non-local Density Functional Theory (NLDFT) [38] was used to calculate the volume of pores smaller than 1 nm (V_<1nm_), volume of micropores (V_mic_), volume of mesopores (V_mes_), total pore volume (V_t_), and pore size distribution (PSD). The surface area was calculated BET method. Scanning electron microscopy (SEM) analysis was carried out on a Supra 55 VP instrument (Zeiss, Portland, OR, USA) with an acceleration voltage of 5 kV.

#### 3.2.2. Surface Chemistry and Bulk Chemical Features

Potentiometric titration (PT) measurements were carried out on a Titrando 888 automatic titrator (Metrohm, Riverview, FL, USA). The equilibrium pH was collected after adding 0.01 mL min^−1^ of a titrant as the titrant volumetric standard 0.1 M NaOH was used. The experiments were run in the pH range of 3–10 and the samples were acidified with 0.100 M HCl to reach the initial pH of the suspension ~3. The titration curves were converted into proton binding curves which were used to calculate pK_a_ distribution of the surface species [41,42].

Thermal analyses (TA) coupled with mass spectrometry (Omnistar GCD 320; Pfeiffer Vacuum Inc., Nashua, NH, USA) was carried out using an SDT Q 600 (TA Instruments, New Castle, DE, USA i) in a He atmosphere. The samples we heated up to 1000 °C at the 10 °C min^−1^ and flow rate of 100 mL min^−1^. The results are presented in terms of the differential thermogravimetric (DTG) curves and *m*/*z* thermal profiles. To verify the content of TiO_2_ and its effects on ignition temperature/reactivity, TA experiments were also carried out in air up to 1000 °C (10 °C min^−1^ and flow rate of 100 mL min^−1^).

The elements present on the surface of the carbons studied as well as their chemical state were identified by X-ray photoelectron spectroscopy (XPS) analysis. A PHI 5000 Versaprobe II spectrometer (Physical Electronics Inc., Chanhassen, MN, USA) was used with Al Kα X-ray radiation (1486.6 eV) as the excitation source. High resolution spectra were recorded at a take-off angle of 45° by using a concentric hemispherical analyzer operating in constant-pass-energy mode at 29.35 eV, with a 200 µm diameter analysis area.

The X-ray diffraction (XRD) spectra were collected on a PANalytical X’Pert X-ray diffractometer (Phillips, Andover, MA, USA) using CuK_α_ (40 kV, 40 mA) radiation. The XRD patterns were recorded in the 2-theta (2θ) range from 5° to 70°, in steps of 0.02° and counting time 0.5 s per step, at room temperature. It is well-known that anatase and rutile are the two main crystalline structures in TiO_2_ P25 [13]. Thus, the influence of the sulfur doping upon the interaction of carbons with the crystalline structures of TiO_2_ was verified from the integration of the intensities of all the anatase and rutile peaks detected in the range 5–75 ° according to the method described elsewhere [13]. The standard deviation of the results obtained using this approach is ~2%.

#### 3.2.3. Optical Features/Band Gap Estimation

UV-visible diffuse reflectance spectra between 200 and 800 nm were measured on a Cary 500 spectrometer (Varian, Palo Alto, CA, USA) with an integrating sphere. The reflectance spectra were converted to Kubelka-Munk function $f\left(R\right)\;=\;\frac{{\left(1\;-\;R\right)}^{1/2}}{2R}$ to construct Tauc plots used to obtain the optical band gaps.

## 4. Conclusions

Summarizing, the results of structural and chemical analyses indicate some significant interactions between TiO_2_ and the carbon phases in the prepared hybrids. Reduced sulfur species seem to be the most involved in these interactions. Even though only physical mixing at ambient conditions was used to synthesize our hybrids, the changes in porosity and chemistry are visible and it is in accordance with the changes detected when the hybrids of sulfur doped carbons with graphite oxide rich in oxygen were formed [43]. These observed changes might be the result of the photoactivity both phases promoting the chemical reactions at the interface. As a result of the approach used, not only new chemistry was formed but also the porosity was significantly affected. Contrary to results reported [44], the band gap increased slightly for the hybrids moving their activity more to the UV region. A decrease in the volume of small pores might affect/limit the adsorption of pollutants and thus their concentration on the surface in a rather negative way. Nevertheless, taking into consideration the high visible light activity of S-doped carbons discussed elsewhere [26,27], building the hybrids or composites with TiO_2_ active in both Vis and UV might enrich the photocatalytic behavior of TiO_2_ not only by the addition of the highly porous and photoactive phase but also by formations of a new interface.

## Figures and Tables

**Table 1:** Content of elements on the surface (in italic; in at. %) and the results of the deconvolution of C 1s, O 1s, S 2p and Ti 2p^3/2^.

Energy, eV	Bond Assignment	T/BAX	T/BAX-S	T/C1	T/C1-S	T/C2	T/C2-S
C 1*s*		*66.4*	*72.6*	*66.5*	*60.7*	*55.7*	*72.0*
284.8	C-(C, S) (graphitic carbon)	63.1	50.9	49.3	28.4	38.1	57.8
286.1	C-O, C-H (phenolic, alcoholic, etheric)	24.1	35.5	34.4	51.7	38.3	19.3
287.0	C=O (carbonyl or quinone)	10.0	9.3	11.4	15.8	15.6	12.7
288.0	O-C=O (carboxyl or ester)	2.8	4.3	4.9	4.1	4.6	7.8
289.0	π-π ^*^	*---*	*---*	*---*	*---*	3.4	2.4
O 1*s*		*26.1*	*21.9*	*25.7*	*30.0*	*32.4*	*20.2*
530.9	TiO_2_	63.9	53.2	55.9	59.2	60.0	63.1
532.4	O=C/O=S (in carboxyl/carbonyl or sulfoxides/sulfones)	12.8	18.4	14.5	11.7	13.2	20.5
533.5	O-C/O-S (in phenol/epoxy or thioesters/sulfonic)	14.8	19.6	17.6	19.6	18.1	16.4
534.5	-O- (in water or chemisorbed oxygen species)	8.5	8.8	12.0	9.5	8.7	---
S 2*p^3^*^/2^				*0.3*	*0.5*	*1.1*	*1.6*
163.4	Ti-S	---	---	41.5	44.0	20.1	75.0
164.6	R-S-S-, C-S-C (in bisulfides/thiophenes configurations)	---	---	---	---	10.0	8.9
166.8	C-S-O, R_2_-S=O/R-SO_2_-R (in sulfoxides, sulfones)	---	---			13.4	
168.8	Sulfonic acid	---	---	58.5	56.0	56.5	16.1
Ti 2*p^3^*^/2^		*7.5*	*5.* *5*	*7.* *5*	*8.8*	*10.8*	*6.2*
459.5	TiO_2_	100	100	100	100	100	100
